# A Review of Pseudorabies Virus Variants: Genomics, Vaccination, Transmission, and Zoonotic Potential

**DOI:** 10.3390/v14051003

**Published:** 2022-05-09

**Authors:** Zongyi Bo, Xiangdong Li

**Affiliations:** 1 Joint International Research Laboratory of Agriculture and Agri-Product Safety, The Ministry of Education of China, Yangzhou University, Yangzhou 225009, China; zybo@yzu.edu.cn; 2 Jiangsu Co-Innovation Center for the Prevention and Control of Animal Infectious Disease and Zoonoses, College of Veterinary Medicine, Yangzhou University, Yangzhou 225009, China

**Keywords:** pseudorabies virus, variant strain, genomics, vaccination, transmission, zoonosis

## Abstract

Pseudorabies virus (PRV), the causative agent of Aujeszky’s disease, has a broad host range including most mammals and avian species. In 2011, a PRV variant emerged in many Bartha K61-vaccinated pig herds in China and has attracted more and more attention due to its serious threat to domestic and wild animals, and even human beings. The PRV variant has been spreading in China for more than 10 years, and considerable research progresses about its molecular biology, pathogenesis, transmission, and host–virus interactions have been made. This review is mainly organized into four sections including outbreak and genomic evolution characteristics of PRV variants, progresses of PRV variant vaccine development, the pathogenicity and transmission of PRV variants among different species of animals, and the zoonotic potential of PRV variants. Considering PRV has caused a huge economic loss of animals and is a potential threat to public health, it is necessary to extensively explore the mechanisms involved in its replication, pathogenesis, and transmission in order to ultimately eradicate it in China.

## 1. Introduction

Pseudorabies Virus, also called Aujeszky’s disease virus or Suid alphaherpesvirus 1, belongs to genus Varicelloviru, Alphaherpesvirinae subfamily within the family Herpesviridae, and is a double-strand linear DNA with 143 kb and can encode more than 70 proteins [1]. PRV was first documented as the causative pathogen of Aujeszky’s disease in 1902 by a Hungarian veterinarian [2]. Different clinical symptoms of pigs can be induced dependent on their housing stages: infected piglets always show fatal and central nervous system disorders, fattening pigs have respiratory symptoms with low mortality, and pregnant sows have abortions with the death of the fetuses [3].

The first case report of PRV in China could be traced back to 1947 in a domestic cat [4]. After that, PRV circulated in many swine herds of China due to the lack of an available PRV vaccine. The Bartha vaccine strain, attenuated through many passages of a virulent strain in culture cells and embryos, was introduced into China in the 1970s, and it provided an ideal protection effect to the early prevalent strains (classical strains) in China [5,6,7]. In late 2011, PRV occurred in many Bartha K61-vaccinated swine herds [8]. After viral isolation and genome sequencing, the results showed that the newly emerging PRV clustered in an independent branch from the previously isolated strains in China [9,10]. To differentiate it from the classical strains isolated before 2011 in China, the newly isolated PRV was designated as a PRV variant.

Though pigs are the only reservoir of PRV, it can infect many species of animals, including sheep, dogs, foxes, tigers, bears, etc. [11,12]. Previously, experimental studies in nonhuman primates showed that rhesus monkeys and marmosets are susceptible to PRV infection, while other higher-order primates, such as chimpanzees and humans are not susceptible to PRV infection [13]. Most recently, more than 20 human severe pseudorabies encephalitis cases were reported [14]. All these patients infected with PRV variants had close contact with pigs which suggested pigs might be the etiological source of PRV for human infection. So, it is time to pay attention to the public threat induced by PRV variants and the transmission of it between different species of animals and human beings.

In this review, we will briefly summarize the studies of PRV variants from the aspects of its outbreak, genomic characteristics, vaccine development, transmission in different animals, and its significance for public health.

## 2. The Outbreaks and the Genomic Evolution of PRV Variants

In late 2011, a large-scale occurrence of severe disease with anorexia, neurologic symptoms, high fever, and respiratory distress in piglets, and a high percent of abortion in sows happened in many swine farms of China. Pathological examination showed gross lesions in the lungs, and yellow-white necrosis in the kidneys. Through multiple kinds of diagnostic methods, including ELISA, PCR, viral isolation, immunohistochemical staining, and gene sequencing, the PRV variant was finally recognized as the causative pathogen for these severe clinical diseases [8,15].

**Figure 1:**
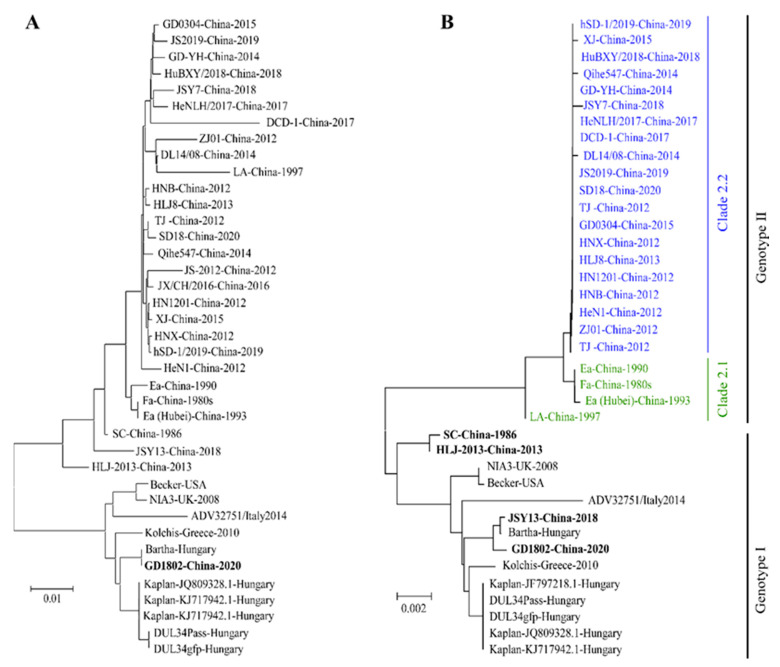
The phylogenetic analysis of PRV full-length genome sequences and gC sequences. (**A**) Phylogenetic analysis of PRV complete genome sequences. (**B**) Phylogenetic analysis of PRV gC gene sequences. Both maximum likelihood (ML) trees were constructed by using MEGA X software.

After viral isolation and sequencing, there is a large sequence divergence in newly isolated PRV strains from previously classical PRV strains. Here, we constructed a maximum likelihood (ML) phylogenetic tree of 39 strains (Supplemental Table S1) of PRV full-length sequences which have been extracted from NCBI. The results showed that all PRV strains can be phylogenetically clustered into two groups. All foreign strains out of China were clustered into the same group, and nearly all strains isolated from China are located in an independent group with them (Figure 1A). Interestingly, the PRV GD1802 strain, isolated in China in 2018, is clustered into the same group with the strains from foreign countries. We infer that the GD1802 strain might be delivered into China from foreign countries through the introduction of pigs. In addition, when compared with classical strains, such as Ea and Fa, PRV strains isolated after 2011 are located in a relatively dependent branch with them. The gC gene is a major gene which has been commonly used for the phylogenetic analysis of PRV due to its high variability [16,17]. We also analyzed gC gene sequences of these reported PRV strains. As shown in Figure 1B, the gC-based phylogenetic tree reveals that PRV can be phylogenetically divided into two groups, designated as genotype I and genotype II, and genotype II can be further divided into two clades, clade 2.1 and clade 2.2. Clade 2.1 mainly comprises the strains isolated before 2011, and nearly all PRV variants are located at clade 2.2.

Recombination contributes a lot to the genomic divergence of many viruses, such as African swine fever virus [18], porcine reproductive and respiratory syndrome virus [19], classical swine fever virus [20], porcine circovirus [21], and porcine epidemic diarrhea virus [22]. As for PRV, the recombination between different strains has been reported in vivo and vitro [23,24]. So, it is interesting to explore whether the evolution of PRV in China also has a relationship with recombination. As shown in Figure 1B, we found that there are four Chinese-origin strains located at the genotype I cluster, including SC, HLJ-2013, JSY13, and GD1802 strains. Except for GD1802, which might be introduced abroad, the other three strains all have a relationship with recombination. Ye et al. found that SC was a recombinant of an endemic Chinese strain and a Bartha-vaccine-like strain [25]; Bo et al. found that the JSY13 strain was a recombination of the PRV variant JSY7 strain and the Bartha K61 vaccine strain [26]; Liu et al. found that the HLJ-2013 strain is probably a recombination of three origins: a yet unknown parent strain, a European-origin strain, and a Chinese-origin strain [27]. Besides these, Huang et al. found that the FJ62 variant strain was the recombination between the PRV genotype I strain from wild boar and genotype II strain from domestic pig [28]. These reports demonstrated that recombination plays an important role in the evolution of PRV in China.

**Figure 2:**
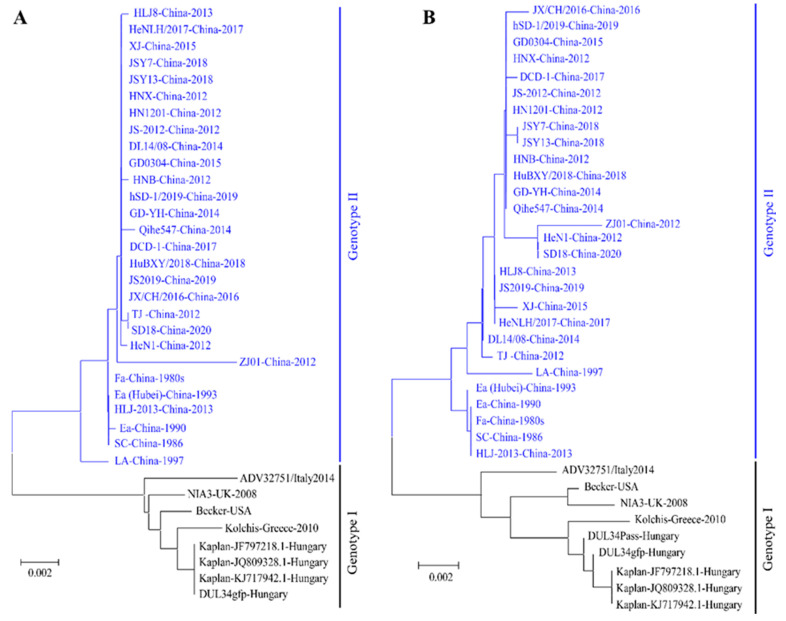
The phylogenetic analysis of PRV gI and gE genes. (**A**) Phylogenetic analysis of PRV gI gene sequences. (**B**) Phylogenetic analysis of PRV gE gene sequences. Both maximum likelihood (ML) trees were constructed by using MEGA X software.

It was reported that the virulence of PRV variants were higher than the classical strains which were isolated before 2011. So, whether there is a difference in the major virulence-determining genes between PRV variants and classical strains warrants investigation. Therefore, we analyzed gI and gE genes, which are major virulence-determining genes of PRV [29]. The phylogenetic analysis showed that all PRV strains can be divided into two classes, as shown in Figure 2A (gI) and Figure 2B (gE). Compared with PRV from foreign countries or the classical strains isolated in China, there are some typical mutations, insertions, and deletions in the several virulence-determining genes, and non-coding sequences of PRV variants, such as two Aspartate (Asp, D) insertions in gE protein [26,30,31].

The biological functions and meanings of these insertions or deletions in PRV variant strains are yet to be explored. Previously, a study showed that the exchange of gB genes contributes to an immunogenic difference between PRV variant JS-2012 and Bartha-K61, which indicated the gB protein of the PRV variant has the ability to evade the neutralization antibody induced by the Bartha strain [32]. The gC gene, a main host receptor binding protein of PRV, can bind to heparan sulfate proteoglycans in the extracellular matrix. Whether the mutations in the gC gene influence the cell entry stage of PRV and viral transmission among different kinds of tissues, animals, and even human beings is rarely studied. As the PRV variant is more virulent than classical PRV strains, whether the mutations in TK, gI, and gE virulence-determining genes are related to the increased virulence of PRV variants also needs to be addressed in the further studies.

## 3. PRV Variant-Based Vaccines Are on the Way

As the PRV variant has been circulating in many Bartha K61-immunized pig farms, researchers tried to explore whether the Bartha K61 strain could provide full protection against PRV variants. An et al. used the PRV variant HeN1 strain to challenge the Bartha K61-immunized pigs and sheep, the results showed that HeN1-challenged pigs showed fever and loss of appetite, while no deaths were found; the experiment on sheep showed that the Bartha K61 strain provided full protection against PRV classical SC strain, but two out of four Bartha K61-immunized sheep challenged with the PRV variant HeN1 strain had clinical signs and died [15]. Luo et al. challenged the Bartha K61-immunized sheep with the PRV variant TJ strain and found that the Bartha K61 vaccine cannot provide complete protection [33]. These experiments demonstrated that the Bartha K61 vaccine can hardly provide full protection against the PRV variant.

Besides the imported Bartha K61 vaccine strain, there are several other licensed PRV live attenuated and inactivated vaccine strains that are based on the PRV strains isolated in China. One of them is the SA125 strain, which is a gE/gI/TK gene-deleted strain based on the cattle-origin Fa strain, and it is the first licensed PRV vaccine in China [34]. Another is the PRV gE/gI/TK gene-deleted HB-2000 strain, which is based on the Ea strain. Besides these two live attenuated vaccines, there is another licensed PRV HB-98 inactivated vaccine which deletes TK/gG genes based on the Ea strain. The existence of the gE gene in the HB-98 vaccine makes it impossible to differentiate the immunized from field strain-infected pigs [35]. Now, there is no direct evidence whether these licensed Chinese-origin PRV strains have better protection than the Bartha K61 vaccine strain to the PRV variant.

The PRV variant has been circulating in China for more than 10 years, and many vaccine candidates that are based on PRV variants have been reported by different research groups. Wang et al. deleted the gE gene of a PRV variant TJ strain and used it to immunize pigs; the pigs had no clinical signs when they were challenged with a PRV variant strain [36]. Wang et al. constructed a TK and gE gene-deleted PRV variant, AH02LA, which could stop the viral shedding, and no clinical signs were observed after being challenged with the PRV variant. By contrast, Bartha K61-immunized pigs showed some mild clinical signs and had viral shedding [37]. Wang et al. used the bacterial artificial chromosome (BCA) manipulation method to construct a PRV variant AH02LA gE gene-deleted strain and found it can provide complete clinical protection against the challenge of the PRV variant [38]. Besides the above-mentioned experimental vaccines, several other PRV variant-based vaccine candidates which have double or triple deletions of TK, gE, or gI genes, also showed complete protection against the challenge of PRV variants [39,40,41]. Till now, there are two licensed PRV variant-based vaccines. One is the PRV C strain, a gI/gE/Us9/Us2 naturally deleted strain that was isolated in China in 2011 and licensed in 2017 [42]. Another is a gE-deleted inactivated vaccine (HN1201-ΔgE), which was certificated in 2019 and could induce a high neutralization antibody titer against both PRV classical and variant strains.

Despite the inactivated and live attenuated vaccines, several other types of vaccines including a subunit vaccine and nucleic vaccine, could also be the alternative for the development of an effective PRV variant vaccine. Among 11 glycoproteins PRV encoded, gB, gC, and gD are the main immunogenetic antigens that can induce neutralization antibodies [7,43]. Previously, Wang et al. used the baculovirus system to express the PRV variant gB protein and challenged the immunized pigs with PRV variant HN1201 strain. The results showed that the gB-based vaccine can provide full protection for the PRV variant [44]. In another report, gB, gC, and gD proteins of the PRV variant were separately expressed using the baculovirus expression system working as subunit vaccine candidates. Pigs were then immunized with each single protein twice and challenged with the PRV variant HNLH strain. The results showed that the survival rates of gB-, gC-, and gD-vaccinated pigs were 100%, 50%, and 87.5%, respectively [45].

A DNA vaccine can mimic the natural infection in which the immunized antigens could be presented in both major histocompatibility complex classes I and II settings [46]. Previously, E.M.A. van Rooij et al. vaccinated pigs with the plasmids expressing the main immunogenetic antigens gB, gC, and gD, then challenged the immunized pigs with PRV. The results showed that plasmid DNA encoding gB induced the strongest cell-mediated immune responses, whereas plasmid DNA encoding gD induced the strongest neutralizing antibody responses [47]. Furthermore, Hyun A Yoon et al. compared the intramuscular (i.m.) and intranasal (i.n.) immunization routes by immunizing mice with the plasmid that encoded gB. The results showed that immunization can induce the mucosal immunity via the i.n. route. However, it only induced a low IgG response which cannot protect the mice against the challenge of PRV [48].

A messenger RNA (mRNA)-based vaccine that encodes the immunogenetic antigen of pathogens provides a new vaccine developing platform for multiple viruses [49,50]. As PRV can encode several immunogenetic antigens that can induce the neutralization antibodies, mRNA might be a candidate for PRV vaccine development. Jiang et al. developed a PRV-XJ variant strain gD gene-based mRNA vaccine which was formulated via mRNA encapsulated in the liposomes. Compared with the DNA vaccine that encoded PRV gD protein in a recombinant plasmid pVAX-gD, both mRNA and pVAX-gD plasmids induced a high neutralization antibody titer and antigen-specific B and T cell response in mice. Finally, the PRV variant challenge experiment was performed, and the results showed that one mouse in ten which was immunized with the mRNA vaccine died, whereas there was no death in the pVAX-gD plasmid-vaccinated group [51]. These results demonstrated that multiple types of vaccines besides inactivated and live attenuated vaccines, show protection against the challenge of PRV, and gB and gD proteins play an important role among these major immunogenetic proteins.

Despite many reports showing that the Bartha K61 vaccine cannot provide full protection against PRV variants, there are still several studies showing that Bartha K61 can provide complete protection to pigs against the PRV variant [7]. Previously, Zhou et al. used and then challenged Bartha K61-immunized growing pigs with the PRV variant XJ5 strain. The results showed that all immunized/challenged pigs survived, while the unimmunized pigs all died [52]. Another report also showed that Bartha K61-immunized pigs had no death under the challenge of the PRV variant AH02LA strain [53]. Although there were some mild clinical signs in immunized/challenged growing pigs, no death was found in the above two studies, which demonstrates that the Bartha K61 vaccine strain can provide protection for pigs against the PRV variant challenge. However, as the PRV variant could lead to the deaths of newborn piglets, further experiments still need to be conducted to confirm whether piglets are resistant to the challenge of the PRV variant because they are Bartha K61-immunized or because they got maternal antibodies form the immunized sows.

## 4. The Pathogenicity of PRV Variant to Different Species of Vertebrates

**Figure 3:**
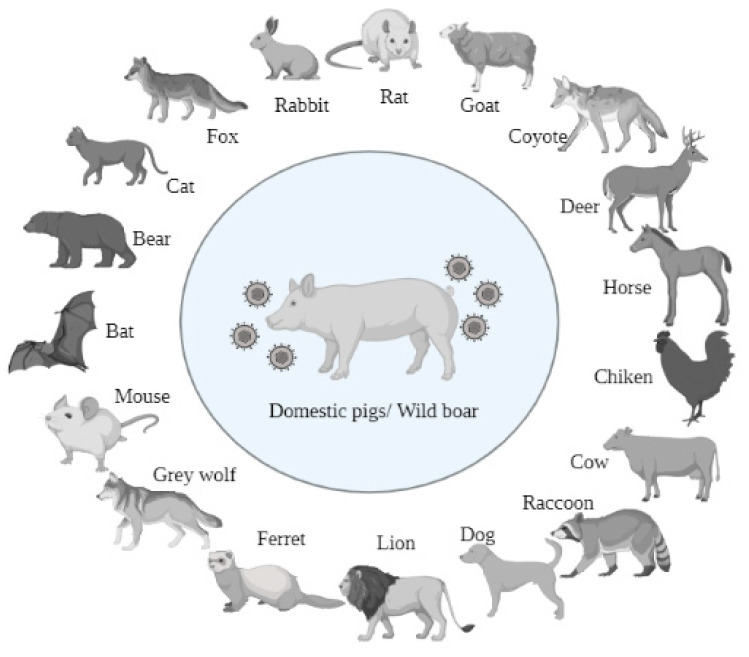
The reported animals that can be infected by PRV.

Although the natural reservoir for PRV is the pig, the first case of PRV was reported in cattle, and the disease was described as “mad itch” [54]. PRV has a broad host range, infecting most mammals and even some avian species. PRV infection can cause different clinical signs dependent on the stages of housing pigs. Besides pigs, PRV infection in other species of animals is always fatal due to the neurologic invasion. Till now, it has been reported that there are about 19 species of animals that can be naturally infected by PRV including domestic/wild pigs [55,56], sheep [57], cats [58], coyotes [59], foxes [60], rats [61], deer [62], bears [63], rabbits [64], dogs [65], horses [66], bats [67], wolves [68], raccoons [69], mice [70], ferrets [71], panthers [72], cattle [73], and chickens [74] (Figure 3). Domestic and wild pigs are the reservoirs for PRV, all other species of animals will die after several days upon the onset of PRV. In the last decade, several animal species including dogs, bovine, mink, foxes, goats, and wolves were found dead due to the infection of PRV variants [75,76,77,78,79]. It is reasonable to infer that some other animal species could be susceptible to PRV variants. The wide host range of PRV can also be demonstrated by its incubation in multiple kinds of cells, such as PK15 cells (Porcine kidney), Vero cells (Green monkey kidney cells), HEp-2 (Human Epithelioma cells), DF-1 cells (Chicken embryo fibroblasts cells), and many other cells. This demonstrates that PRV can bind with its receptor in the surface of these cells, which suggests that PRV might have the possibility to infect multiple kinds of animals.

Besides natural infection, the experimental infections of PRV were also conducted to confirm the susceptibility of different animals, such as domestic pigs [80], wild boars [81], sheep [82], calves [73], dogs [83], cats [84], blue foxes [85], raccoons [86], horses [66], and chickens [74]. The clinical signs after infection mainly include fever, depression, pruritus, self-mutilation, anorexia, neurologic deficits, etc. The viral challenge experiments demonstrated that PRV can be introduced through multiple kinds of routes, including intracranial, intradermal, intramuscular, intranasal, intraocular, intraperitoneal, intratracheal, intravenous, and even foot pads [87]. The animal experiments of PRV can also be performed in some laboratory animals, including mice [88], rats [89], rabbits [90], dogs [90], guinea pigs [91], and rhesus macaques [92]. Due to the wide host range and easy manipulation, PRV can be handled as the model to study many characteristics of herpesvirus, such as molecular biology, pathogenesis, neuroinvasion, and transneuronal spread.

Sometimes the transmission of PRV among different species of animals will lead to the change of PRV pathogenicity. Previously, R E Shope found that PRV was attenuated after passaging through a guinea pig brain [91]. In addition, T F Müller et al. found that PRV strains of wild swine origin might be less virulent than those of domestic pigs [81]. This phenomenon might be explained by the insertions or deletions of multiple segments among different strains, in order to adapt to their host animals.

PRV is an air-borne pathogen. Furthermore, food, water, and excrement can also be intermediate vectors of PRV [93]. The infected pigs or semen are the main origins for PRV infection in swine farms. As for other species of animals, the direct contact of contaminated equipment, or consumption of the infected animals are the main sources for their infection [94]. Thereafter, it is important to keep the animals away from swine farms, especially mice, cats, dogs, etc., in case they are infected with PRV and transmit it to other animals. Furthermore, pig products or byproducts, such as head or offal tissue, which might be polluted by PRV must be kept away from other animals, since they could directly contact or consume them. Finally, surveillance of PRV not only in pigs, but also in other species of animals should be performed, in order to keep PRV out of the susceptible herds.

## 5. The Zoonotic Potential of PRV Variants

It has been known that PRV has a wide host range, and the duplication and translocation of sequences from the left end of the genome to the UL-US junction plays an important role for the growth of PRV in different hosts [95]. It was reported that Nectin-1, a host cellular receptor that can bind with PRV glycoprotein gD, plays an important role in PRV entry. Previous studies reported that PRV gD glycoprotein showed a similar binding ability to human nectin-1 protein [96]. Not only human nectin-1, the protein that comes from bats, dogs, cats, cows, sheep, and several other animals also showed conserved functional amino acids binding with PRV gD, which provides evidence of cross-species infection for PRV [14].

The first case report of humans infected with PRV was in 1914, in which two laboratory technicians were infected with PRV after they were exposed to an infected cat. Afterwards, several PRV cases in humans were reported after they came into contact with cast, dogs, cows, or other domestic animals (Summarized in Table 1). The typical symptoms of these cases mainly include pruritis, pain, fever, swelling, sweating, dysphagia, and aphthous stomatitis. Though these PRV infected cases were reported, the etiological confirmation was not convincing due to the lack of a definitely etiological and serological diagnosis.

In 2017, a swine herder suffered from an eye disease in China. Through next-generation sequencing (NGS), real-time PCR, and phylogenetic analysis, the PRV variant was recognized as the causative agent of the disease [97]. This is the first PRV case in humans reported in China. In the following years, several other PRV infection cases in human beings were reported in China (Table 1). Different from the clinical symptoms reported from other countries, the clinical signs of Chinese patients in these cases were mainly encephalitis and endophthalmitis. Of note, almost all PRV infected patients have contact with swine or other PRV-susceptible animals. Among these incidences, a milestone case attracted our attention because one PRV variant, designated hSD-1/2019, was firstly isolated from the cerebrospinal fluid of a PRV-infected patient [98]. This is the first case in the world where PRV was successfully isolated from humans, which proved that humans are susceptible to PRV.

Of note, the cases of humans infected with PRV have been increasing rapidly in China since 2017. Coincidentally, PRV variants have been the dominant strain in Chinese pig farms since then. Whether PRV variants are more sensitive to humans than the classical PRV warrants further investigation. Among these PRV-infected patients, most of them were reported as having swine-related occupations or had close contact with other infected animals. Furthermore, several patients have injuries to their fingers or other places. Therefore, it is necessary to carry out skin protection for people who have close contact with swine. Till now, all infected humans recovered completely, and the clinical and neurological signs disappeared, though sometimes the clinical signs last for one to several months. No patients died from PRV infection in the above cases. However, similar to other herpesviruses, PRV can induce latent and lytic replication in pigs, and the infected pigs will carry PRV for a long time. Whether the recovered patients still carry PRV is unknown.

## 6. Discussion and Perspectives

In this review, we summarized the biological characteristics of PRV variants, which have antigenic variation and a higher virulence compared with classical PRV strains in China. Since 2011, PRV variants have become the dominant strains in China and caused huge economic losses for the swine industry. There is still dispute over whether the Bartha K61 vaccine can provide full protection against the PRV variant [52,113]. However, PRV variants happened in many Bartha K61-immunized swine farms, and the phylogenetic analysis showed that PRV variants have a large sequence divergence with the Bartha K61 strain. Therefore, more effective vaccines based on local PRV variants need to be developed. Moreover, multiple kinds of vaccines, including a live attenuated vaccine, inactivated vaccine, subunit vaccine, DNA vaccine, and mRNA vaccine have the potential to provide full protection for pigs against PRV variants. Upon the design of PRV vaccines, it is necessary to follow the strategy of DIVA, which has helped to eradicate PRV in many countries [114].

The serological detection of PRV gE antibodies is always used to evaluate the status of PRV field strain infection, due to the application of gE-deleted PRV vaccines. One study reported that PRV positive rates have decreased to 18.12% in 2020 from 38.20% in 2018 after screening 19,292 pig serum samples by using PRV gE ELISA [115]. Another report analyzed the gE antibodies from 256,326 serum samples, which were collected from 29 provinces in China from 2011 to 2021. The results showed that the average positive rate was 29.87% [31]. Zheng et al. collected 4708 pig serum samples from Henan province during 2018–2019 and found the positive rate of gE antibodies was 30.14% (1419/4708) [116]. Lin et at. collected a total of 18,138 serum samples from 808 PRV-vaccinated pig farms during 2016–2020 in Hunan province and detected the presence of gE-specific antibodies. The results showed that 23.55% (4271/18,138) of the samples were positive for PRV gE-specific antibodies [117]. All these results demonstrate that the infection of the PRV field strain is still very common in Chinese swine farms, though nearly all of them have used PRV vaccines. Therefore, strict biosecurity measures, feeding strategies, daily managements, diagnostic methods, and effective vaccination should be jointly performed in swine farms to decrease the infection of the PRV field strain.

Right now, with the increased number of human cases caused by PRV infection, the exact mechanisms involved in its transmission from animals to human beings are still unknown. One explanation is that the “one health” concept is recognized by more and more researchers, and the interdisciplinary communication is more frequent than before, so the human cases with encephalitis and neurological symptoms, which might be induced by PRV, could be jointly diagnosed by doctors and veterinarians. Another possibility is that the evolution of the PRV variant makes it more susceptible to humans as PRV entry-related proteins gD or gC might have a higher binding ability to its receptor in human cells than before. In contrast, a study showed that 455 persons who participated in heroic self-incubation with PRV via intracutaneous and subcutaneous methods showed no clinical signs, which demonstrated that the infection of PRV in humans was occasionally asymptomatic [99]. We hypothesize that the infection of PRV in humans might be related to the immune system of people, and immunocompromised patients might be more susceptible to PRV. Till now, the only consolatory thing is that there is no human-to-human PRV transmission case. More studies about the mechanisms evolved in the transmission of PRV from infected animals to humans still need to be explored.

Due to the lack of effective PRV variant vaccines, many researchers have focused on exploring the available compounds that can inhibit the proliferation of PRV in vitro and in vivo. Among them, two kinds of compounds attract people’s attention. One is traditional Chinese herbal medicines, which have a long history of anti-virus effects, but their cellular targets and working mechanisms are unclear. For example, Germacrone, which is extracted from Rhizoma Curcuma, was able to inhibit the proliferation of PRV in the early phase of the PRV replication cycle [118]. Another kind is synthetic chemical compounds, which have a relatively clear cellular target and whose functions are comparatively clear. For example, it was demonstrated that meclizine, a class of H1-antihistamine, could inhibit the replication of PRV at its entry and release stages [119]. The exploration of anti-PRV compounds will contribute to the therapy of PRV infection, especially for the infection of human beings.

Because of the existence of multiple non-essential genes, PRV can be engineered as the vector to express foreign antigens derived from other pathogens. For example, Qiu et al. used PRV as the vector to express the GP5 protein of porcine reproductive and respiratory syndrome virus, and the recombinant virus provided an ideal protection against the challenge of PRRSV [120]. Other proteins, such as the HA gene of the H3N2 subtype of swine influenza virus, and the S protein of porcine epidemic diarrhea virus, were also expressed by PRV, which provided protection to the immunized pigs [121,122]. The advantage of these vectored vaccines are that they provide protection against the infections of other pathogens besides PRV.

In conclusion, as the PRV variant has caused a huge economic loss for the pig industry in China and is a potential threat to public health, it is necessary to pay more attention to the detection and isolation of the PRV variant in different animals, in order to study its epidemiological characteristics. At the same time, it is urgent to develop more safe and efficient DIVA vaccines based on the PRV variants. Furthermore, it is also important to deeply explore the interactions between the PRV infection and host responses, which will not only help to clarify the mechanisms involved in PRV proliferation and pathogenesis, but also contribute to the development of PRV vaccines and antiviral drugs. Most importantly, since PRV has the possibility to infect human beings, researchers need to pay more attention to its cross-species transmission and screen the patients that are suspected of being infected by PRV, especially those who have swine-related occupations.

## Figures and Tables

**Table 1:** PRV infection cases in humans.

Year	Numbers	Symptoms	Contacted Animals	Ref.
1914	2	Swelling, reddening, intense itching	Cat	[99]
1940	2	Pruritis, erythema, and pain around the wound	Dog	[99]
1963	2	Throat pain, weakness in the legs,	Dog	[99]
1983	1	Pain in togue, hypersalivation, dysphagia, headache, arthralgia	Cat	[100]
1986	2	Tension in the mouth, nose, and throat; perception of strange smells and taste	Cat, or other domestic animals	[100]
1992	6	Pruritus in the palms, lower and upper arms, shoulders, and back	Cow	[101]
2017	1	Endophthalmitis, fever, headaches	Pig	[97]
2018	4	Encephalitis	Pig	[102]
2019	5	Encephalitis	Pig	[103]
2019	1	Encephalitis	Pig	[104]
2019	1	Encephalitis, fever, headache, seizure	unknown	[105]
2019	1	Encephalitis	Pig	[106]
2020	1	Encephalitis	Pig	[107]
2020	6	panencephalitis	Pig	[108]
2021	1	Retinitis	Pig	[109]
2021	1	Encephalitis, retinal vasculitis, fever	Pig	[110]
2021	1	Encephalitis, Endophthalmitis	Pig	[111]
2022	2	Encephalitis, seizures, endophthalmitis	Pig	[112]

## Data Availability

All data generated or analyzed during this study are included in the published article.

## Supplementary Materials

The following supporting information can be downloaded at: https://www.mdpi.com/article/10.3390/v14051003/s1, Table S1: The genomic information of 39 strains of PRV.

## References

[1] Pomeranz L.E., Reynolds A.E., Hengartner C.J. (2005). Molecular Biology of Pseudorabies Virus: Impact on Neurovirology and Veterinary Medicine. Microbiol. Mol. Biol. Rev..

[2] Wittmann G., Rziha H.-J. (1989). Aujeszk™ s Disease (Pseudorabies) in Pigs. Herpesvirus Diseases of Cattle, Horses, and Pigs.

[3] Mettenleiter T.C. (2000). Aujeszky’s disease (pseudorabies) virus: The virus and molecular pathogenesis—State of the art, June 1999. Vet. Res..

[4] Yuan Q.Z., Wu Y.X., Li Y.X., Li Z.R., Nan X. (1983). The pseudorabies vaccination research. I: Pseudorabies attenuated vaccine research. Chin. J. Prev. Vet. Med..

[5] Lomniczi B., Kaplan A.S., Ben-Porat T. (1987). Multiple defects in the genome of pseudorabies virus can affect virulence without detectably affecting replication in cell culture. Virology.

[6] Sun Y., Luo Y., Wang C.-H., Yuan J., Li N., Song K., Qiu H.-J. (2016). Control of swine pseudorabies in China: Opportunities and limitations. Vet. Microbiol..

[7] Freuling C.M., Müller T.F., Mettenleiter T.C. (2017). Vaccines against pseudorabies virus (PrV). Vet. Microbiol..

[8] Yu X., Zhou Z., Hu D., Zhang Q., Han T., Li X., Gu X., Yuan L., Zhang S., Wang B. (2014). Pathogenic Pseudorabies Virus, China, 2012. Emerg. Infect. Dis..

[9] He W., Auclert L.Z., Zhai X., Wong G., Zhang C., Zhu H., Xing G., Wang S., He W., Li K. (2019). Interspecies Transmission, Genetic Diversity, and Evolutionary Dynamics of Pseudorabies Virus. J. Infect. Dis..

[10] Zhai X., Zhao W., Li K., Zhang C., Wang C., Su S., Zhou J., Lei J., Xing G., Sun H. (2019). Genome Characteristics and Evolution of Pseudorabies Virus Strains in Eastern China from 2017 to 2019. Virol. Sin..

[11] Laval K., Enquist L.W. (2020). The Neuropathic Itch Caused by Pseudorabies Virus. Pathogens.

[12] Müller T., Hahn E.C., Tottewitz F., Kramer M., Klupp B.G., Mettenleiter T.C., Freuling C. (2011). Pseudorabies virus in wild swine: A global perspective. Arch. Virol..

[13] Enquist L.W. (1999). Life beyond eradication: Veterinary viruses in basic science. Arch. Virol. Suppl..

[14] Wong G., Lu J., Zhang W., Gao G.F. (2019). Pseudorabies virus: A neglected zoonotic pathogen in humans?. Emerg. Microbes Infect..

[15] An T.-Q., Peng J.-M., Tian Z.-J., Zhao H.-Y., Li N., Liu Y.-M., Chen J.-Z., Leng C.-L., Sun Y., Chang D. (2013). Pseudorabies Virus Variant in Bartha-K61–Vaccinated Pigs, China, 2012. Emerg. Infect. Dis..

[16] Ishikawa K., Tsutsui M., Taguchi K., Saitoh A., Muramatsu M. (1996). Sequence variation of the gC gene among pseudorabies virus strains. Vet. Microbiol..

[17] Ye C., Zhang Q.-Z., Tian Z.-J., Zheng H., Zhao K., Liu F., Guo J.-C., Tong W., Jiang C.-G., Wang S.-J. (2015). Genomic characterization of emergent pseudorabies virus in China reveals marked sequence divergence: Evidence for the existence of two major genotypes. Virology.

[18] Zhu Z., Xiao C.-T., Fan Y., Cai Z., Lu C., Zhang G., Jiang T., Tan Y., Peng Y. (2019). Homologous recombination shapes the genetic diversity of African swine fever viruses. Vet. Microbiol..

[19] Zhou L., Kang R., Zhang Y., Yu J., Xie B., Chen C., Li X., Chen B., Liang L., Zhu J. (2019). Emergence of two novel recombinant porcine reproductive and respiratory syndrome viruses 2 (lineage 3) in Southwestern China. Vet. Microbiol..

[20] Chen Y., Chen Y.-F. (2014). Extensive homologous recombination in classical swine fever virus: A re-evaluation of homologous recombination events in the strain AF407339. Saudi J. Biol. Sci..

[21] Wei C., Lin Z., Dai A., Chen H., Ma Y., Li N., Wu Y., Yang X., Luo M., Liu J. (2019). Emergence of a novel recombinant porcine circovirus type 2 in China: PCV2c and PCV2d recombinant. Transbound. Emerg. Dis..

[22] Chen N., Li S., Zhou R., Zhu M., He S., Ye M., Huang Y., Li S., Zhu C., Xia P. (2017). Two novel porcine epidemic diarrhea virus (PEDV) recombinants from a natural recombinant and distinct subtypes of PEDV variants. Virus Res..

[23] Maes R.K., Sussman M.D., Vilnis A., Thacker B.J. (1997). Recent developments in latency and recombination of Aujeszky’s disease (pseudorabies) virus. Vet. Microbiol..

[24] Dangler C.A., Henderson L.M., Bowman L.A., Deaver R.E. (1993). Direct isolation and identification of recombinant pseudorabies virus strains from tissues of experimentally co-infected swine. Am. J. Vet. Res..

[25] Ye C., Guo J.-C., Gao J.-C., Wang T.-Y., Zhao K., Chang X.-B., Wang Q., Peng J.-M., Tian Z.-J., Cai X.-H. (2016). Genomic analyses reveal that partial sequence of an earlier pseudorabies virus in China is originated from a Bartha-vaccine-like strain. Virology.

[26] Bo Z., Miao Y., Xi R., Gao X., Miao D., Chen H., Jung Y.S., Qian Y., Dai J. (2020). Emergence of a novel pathogenic recombinant virus from Bartha vaccine and variant pseudorabies virus in China. Transbound. Emerg. Dis..

[27] Liu H., Shi Z., Liu C., Wang P., Wang M., Wang S., Liu Z., Wei L., Sun Z., He X. (2020). Implication of the Identification of an Earlier Pseudorabies Virus (PRV) Strain HLJ-2013 to the Evolution of Chinese PRVs. Front. Microbiol..

[28] Huang J., Zhu L., Zhao J., Yin X., Feng Y., Wang X., Sun X., Zhou Y., Xu Z. (2020). Genetic evolution analysis of novel recombinant pseudorabies virus strain in Sichuan, China. Transbound. Emerg. Dis..

[29] Ferrari M., Mettenleiter T., Romanelli M., Cabassi E., Corradi A., Mas N.D., Silini R. (2000). A Comparative Study of Pseudorabies Virus (PRV) Strains with Defects in Thymidine Kinase and Glycoprotein Genes. J. Comp. Pathol..

[30] Fan J., Zeng X., Zhang G., Wu Q., Niu J., Sun B., Xie Q., Ma J. (2016). Molecular characterization and phylogenetic analysis of pseudorabies virus variants isolated from Guangdong province of southern China during 2013–2014. J. Vet. Sci..

[31] Tan L., Yao J., Yang Y., Luo W., Yuan X., Yang L., Wang A. (2021). Current Status and Challenge of Pseudorabies Virus Infection in China. Virol. Sin..

[32] Yu Z.-Q., Tong W., Zheng H., Li L.-W., Li G.-X., Gao F., Wang T., Liang C., Ye C., Wu J.-Q. (2017). Variations in glycoprotein B contribute to immunogenic difference between PRV variant JS-2012 and Bartha-K61. Vet. Microbiol..

[33] Luo Y., Li N., Cong X., Wang C.-H., Du M., Li L., Zhao B., Yuan J., Liu D.-D., Li S. (2014). Pathogenicity and genomic characterization of a pseudorabies virus variant isolated from Bartha-K61-vaccinated swine population in China. Vet. Microbiol..

[34] Ling Z., Wan-Zhu G., Zhi-Wen X. (2004). Fluctuant Rule of Colostral Antibodies and the Date of Initial Immunization for the Piglet from Sows Inoculated with Pseudorabies Virus Gene-deleted Vaccine SA215. Chin. J. Vet. Sci..

[35] He Q.G., Chen H.C., Fang L.R., Wu B., Liu Z.F., Xiao S.B., Jin M.L. (2006). The Safety, Stablization and Immunogenicity of Double Gene-negative Mutant of Pseudorabies Virus Strain (PrV HB-98). Chin. J. Vet. Sci..

[36] Wang C.-H., Yuan J., Qin H.-Y., Luo Y., Cong X., Li Y., Chen J., Li S., Sun Y., Qiu H.-J. (2014). A novel gE-deleted pseudorabies virus (PRV) provides rapid and complete protection from lethal challenge with the PRV variant emerging in Bartha-K61-vaccinated swine population in China. Vaccine.

[37] Wang J., Song Z., Ge A., Guo R., Qiao Y., Xu M., Wang Z., Liu Y., Zheng Y., Fan H. (2018). Safety and immunogenicity of an attenuated Chinese pseudorabies variant by dual deletion of TK&gE genes. BMC Vet. Res..

[38] Wang J., Guo R., Qiao Y., Xu M., Wang Z., Liu Y., Gu Y., Liu C., Hou J. (2016). An inactivated gE-deleted pseudorabies vaccine provides complete clinical protection and reduces virus shedding against challenge by a Chinese pseudorabies variant. BMC Vet. Res..

[39] Gu Z., Dong J., Wang J., Hou C., Sun H., Yang W., Bai J., Jiang P. (2015). A novel inactivated gE/gI deleted pseudorabies virus (PRV) vaccine completely protects pigs from an emerged variant PRV challenge. Virus Res..

[40] Tong W., Li G., Liang C., Liu F., Tian Q., Cao Y., Li L., Zheng X., Zheng H., Tong G. (2016). A live, attenuated pseudorabies virus strain JS-2012 deleted for gE/gI protects against both classical and emerging strains. Antivir. Res..

[41] Hu R.-M., Zhou Q., Song W.-B., Sun E.-C., Zhang M.-M., He Q.-G., Chen H.-C., Wu B., Liu Z.-F. (2015). Novel pseudorabies virus variant with defects in TK, gE and gI protects growing pigs against lethal challenge. Vaccine.

[42] Gao J.F., Lai Z., Shu Y.H., Qi S.H., Ma J.J., Wu B.Q., Gong J.P. (2015). Isolation and identification of porcine pseudorabies virus (PRV) C strain. Acta Agric. Shanghai.

[43] Mettenleiter T.C. (1994). Pseudorabies (Aujeszky’s disease) virus: State of the art. August 1993. Acta Vet. Hung..

[44] Wang Y., Wang T., Yan H., Yang F., Guo L., Yang Q., Hu X., Tan F., Xiao Y., Li X. (2015). Research and development of a novel subunit vaccine for the currently circulating pseudorabies virus variant in China. Front. Agric. Sci. Eng..

[45] Zhang T., Liu Y., Chen Y., Wang A., Feng H., Wei Q., Zhou E., Zhang G. (2020). A single dose glycoprotein D-based subunit vaccine against pseudorabies virus infection. Vaccine.

[46] Porter K.R., Raviprakash K. (2017). DNA Vaccine Delivery and Improved Immunogenicity. Curr. Issues Mol. Biol..

[47] Van Rooij E.M., Haagmans B.L., Glansbeek H.L., de Visser Y.E., de Bruin M.G., Boersma W., Bianchi A.T. (2000). A DNA vaccine coding for glycoprotein B of pseudorabies virus induces cell-mediated immunity in pigs and reduces virus excretion early after infection. Vet. Immunol. Immunopathol..

[48] Yoon H.A., Han Y.W., Aleyas A., George J.A., Kim S.J., Kim H.K., Song H.J., Cho J.G., Eo S.K. (2008). Protective immunity induced by systemic and mucosal delivery of DNA vaccine expressing glycoprotein B of pseudorabies virus. J. Microbiol. Biotechnol..

[49] Pardi N., Hogan M.J., Porter F.W., Weissman D. (2018). mRNA vaccines—A new era in vaccinology. Nat. Rev. Drug Discov..

[50] Jackson N.A.C., Kester K.E., Casimiro D., Gurunathan S., DeRosa F. (2020). The promise of mRNA vaccines: A biotech and industrial perspective. NPJ Vaccines.

[51] Jiang Z., Zhu L., Cai Y., Yan J., Fan Y., Lv W., Gong S., Yin X., Yang X., Sun X. (2020). Immunogenicity and protective efficacy induced by an mRNA vaccine encoding gD antigen against pseudorabies virus infection. Vet. Microbiol..

[52] Zhou J., Li S., Wang X., Zou M., Gao S. (2017). Bartha-k61 vaccine protects growing pigs against challenge with an emerging variant pseudorabies virus. Vaccine.

[53] Wang J., Zeng R., Torrents D., Martinez C., Qiao Y., Yiqi G.U., Liu C.J.A.H., Medicine V. (2015). Protection of pseudorabies vaccine (Bartha K61 strain) against pseudorabies virus variant in pigs. Anim. Husb. Vet. Med..

[54] Hanson R.P. (1954). The history of pseudorabies in the United States. J. Am. Vet. Med. Assoc..

[55] Beran G.W., Davies E.B., Arambulo P.V., Will L.A., Hill H.T., Rock D.L. (1980). Persistence of pseudorabies virus in infected swine. J. Am. Vet. Med. Assoc..

[56] Verpoest S., Cay A.B., De Regge N. (2014). Molecular characterization of Belgian pseudorabies virus isolates from domestic swine and wild boar. Vet. Microbiol..

[57] Mocsári E., Szolnoki J., Glávits R., Zsák L. (1989). Horizontal transmission of Aujeszky’s disease virus from sheep to pigs. Vet. Microbiol..

[58] Egberink H.F. (1990). Aujeszky’s disease in dogs and cats. Tijdschr. Voor Diergeneeskd..

[59] Raymond J.T., Gillespie R.G., Woodruff M., Janovitz E.B. (1997). Pseudorabies in Captive Coyotes. J. Wildl. Dis..

[60] Murdoch R.S. (1990). Aujeszky’s disease in foxhounds. Vet. Rec..

[61] Dolivo M., Beretta E., Bonifas V., Foroglou C. (1978). Ultrastructure and function in sympathetic ganglia isolated from rats infected with pseudorabies virus. Brain Res..

[62] Neagari Y., Sakai T., Nogami S., Kaiho I., Katoh C. (1998). Incidence of antibodies in raccoon dogs and deer inhabiting suburban areas. Kansenshogaku Zasshi.

[63] Banks M., Torraca L.S.M., Greenwood A.G., Taylor D.C. (1999). Aujeszky’s disease in captive bears. Vet. Rec..

[64] Goto H., Burger D., Gorham J. (1971). Quantitative studies of pseudirabies virus in mink, ferrets, rabbits and mice. Jpn. J. Vet. Sci..

[65] Guillon J.C., Chirol C., Vallée A., Cordaillat J.C., Beylot J.C. (1968). A focus of Aujeszky’s disease in dogs in the department of Ain. Bull. L’acad. Vet. Fr..

[66] Kimman T.G., Binkhorst G.J., Ingh T.S.V.D., Pol J.M., Gielkens A.L., Roelvink M.E. (1991). Aujeszky’s disease in horses fulfils Koch’s postulates. Vet. Rec..

[67] Reagan R.L., Day W.C., Marley R.T., Brueckner A.L. (1953). Effect of pseudorabies virus (Aujeszky strain) in the large brown bat (Eptesicus fuscus). Am. J. Vet. Res..

[68] Amoroso M.G., Di Concilio D., D’Alessio N., Veneziano V., Galiero G., Fusco G. (2020). Canine parvovirus and pseudorabies virus coinfection as a cause of death in a wolf (Canis lupus) from southern Italy. Vet. Med. Sci..

[69] Thawley D., Wright J. (1982). Pseudorabies virus infection in raccoons: A review. J. Wildl. Dis..

[70] Field H.J., Hill T.J. (1974). The Pathogenesis of Pseudorabies in Mice following Peripheral Inoculation. J. Gen. Virol..

[71] Ohshima K.I., Gorham J.R., Henson J.B. (1976). Pathologic changes in ferrets exposed to pseudorabies virus. Am. J. Vet. Res..

[72] Glass C.M., McLean R.G., Katz J.B., Maehr D.S., Cropp C.B., Kirk L.J., McKeiman A.J., Evermann J.F. (1994). Isolation of pseudorabies (Aujeszky’s Disease) virus from a florida panther. J. Wildl. Dis..

[73] McCracken R.M., McFerran J.B., Dow C. (1973). The Neural Spread of Pseudorabies Virus in Calves. J. Gen. Virol..

[74] Ramachandran S.P., Fraser G. (1971). Studies on the virus of Aujeszky’s disease. J. Comp. Pathol..

[75] Tu L., Lian J., Pang Y., Liu C., Cui S., Lin W. (2021). Retrospective detection and phylogenetic analysis of pseudorabies virus in dogs in China. Arch. Virol..

[76] Cheng Z., Kong Z., Liu P., Fu Z., Zhang J., Liu M., Shang Y. (2020). Natural infection of a variant pseudorabies virus leads to bovine death in China. Transbound. Emerg. Dis..

[77] Lian K., Zhang M., Zhou L., Song Y., Wang G., Wang S. (2020). First report of a pseudorabies-virus-infected wolf (Canis lupus) in China. Arch. Virol..

[78] Liu H., Li X.-T., Hu B., Deng X.-Y., Zhang L., Lian S.-Z., Zhang H.-L., Lv S., Xue X.-H., Lu R.-G. (2017). Outbreak of severe pseudorabies virus infection in pig-offal-fed farmed mink in Liaoning Province, China. Arch. Virol..

[79] Jin H.-L., Gao S.-M., Liu Y., Zhang S.-F., Hu R.-L. (2016). Pseudorabies in farmed foxes fed pig offal in Shandong province, China. Arch. Virol..

[80] Lin J., Li Z., Feng Z., Fang Z., Chen J., Chen W., Liang W., Chen Q. (2020). Pseudorabies virus (PRV) strain with defects in gE, gC, and TK genes protects piglets against an emerging PRV variant. J. Vet. Med Sci..

[81] Müller T.F., Teuffert J., Zellmer R., Conraths F.J. (2001). Experimental infection of European wild boars and domestic pigs with pseudorabies viruses with differing virulence. Am. J. Vet. Res..

[82] Schmidt S.P., Hagemoser W.A., Kluge J.P., Hill H.T. (1987). Pathogenesis of ovine pseudorabies (Aujeszky’s disease) following intratracheal inoculation. Can. J. Vet. Res..

[83] Zhang L., Zhong C., Wang J., Lu Z., Liu L., Yang W., Lyu Y. (2015). Pathogenesis of natural and experimental Pseudorabies virus infections in dogs. Virol. J..

[84] Hagemoser W.A., Kluge J.P., Hill H.T. (1980). Studies on the pathogenesis of pseudorabies in domestic cats following oral inoculation. Can. J. Comp. Med..

[85] Quiroga M.I., Vázquez S., López-Peña M., Guerrero F., Nieto J.M. (1995). Experimental Aujeszky’s Disease in Blue Foxes (Alopex lagopus. J. Vet. Med. Ser. A.

[86] Kirkpatrick C.M., Kanitz C.L., McCrocklin S.M. (1980). Possible Role of wild mammals in transmission of pseudorabies to swine. J. Wildl. Dis..

[87] Sehl J., Teifke J.P. (2020). Comparative Pathology of Pseudorabies in Different Naturally and Experimentally Infected Species—A Review. Pathogens.

[88] Laval K., Vernejoul J.B., Van Cleemput J., Koyuncu O.O., Enquist L.W. (2018). Virulent Pseudorabies Virus Infection Induces a Specific and Lethal Systemic Inflammatory Response in Mice. J. Virol..

[89] Rassnick S., Enquist L.W., Sved A.F., Card J. (1998). Pseudorabies Virus-Induced Leukocyte Trafficking into the Rat Central Nervous System. J. Virol..

[90] Olander H.J., Saunders J., Gustafson D., Jones R. (1966). Pathologic Findings in Swine Affected with a Virulent Strain of Aujeszky’s Virus. Pathol. Vet..

[91] Shope R.E. (1933). Modification of the pathogenicity of pseudorabies virus by animal passage. J. Exp. Med..

[92] Hurst E.W. (1936). Studies on pseudorabies (infectious bulbar paralysis, mad itch). J. Exp. Med..

[93] Li H., Liang R., Pang Y., Shi L., Cui S., Lin W. (2020). Evidence for interspecies transmission route of pseudorabies virus via virally contaminated fomites. Vet. Microbiol..

[94] Hahn E., Page G., Hahn P., Gillis K., Romero C., Annelli J., Gibbs E. (1997). Mechanisms of transmission of Aujeszky’s disease virus originating from feral swine in the USA. Vet. Microbiol..

[95] Reilly L.M., Rall G., Lomniczi B., Mettenleiter T.C., Kuperschmidt S., Ben-Porat T. (1991). The ability of pseudorabies virus to grow in different hosts is affected by the duplication and translocation of sequences from the left end of the genome to the UL-US junction. J. Virol..

[96] Zhang N., Yan J., Lu G., Guo Z., Fan Z., Wang J., Shi Y., Qi J., Gao G.F. (2011). Binding of herpes simplex virus glycoprotein D to nectin-1 exploits host cell adhesion. Nat. Commun..

[97] Ai J.-W., Weng S.-S., Cheng Q., Cui P., Li Y.-J., Wu H.-L., Zhu Y.-M., Xu B., Zhang W.-H. (2018). Human Endophthalmitis Caused By Pseudorabies Virus Infection, China, 2017. Emerg. Infect. Dis..

[98] Liu Q., Wang X., Xie C., Ding S., Yang H., Guo S., Li J., Qin L., Ban F., Wang D. (2021). A Novel Human Acute Encephalitis Caused by Pseudorabies Virus Variant Strain. Clin. Infect. Dis..

[99] Skinner G., Ahmad A., Davies J. (2001). The infrequency of transmission of herpesviruses between humans and animals; postulation of an unrecognised protective host mechanism. Comp. Immunol. Microbiol. Infect. Dis..

[100] Avak S., Bienzle U., Feldmeier H., Hampl H., Habermehl K.-O. (1987). Pseudorabies in man. Lancet.

[101] Anusz Z., Szweda W., Popko J., Trybała E. (1992). Is Aujeszky’s disease a zoonosis?. Prz. Epidemiol..

[102] Zhao W.L., Wu Y.H., Li H.F., Li S.Y., Fan S.Y., Wu H.L., Li Y.J., Lü Y.L., Han J., Zhang W.C. (2018). Clinical experience and next-generation sequencing analysis of encephalitis caused by pseudorabies virus. Zhonghua Yi Xue Za Zhi.

[103] Yang X., Guan H., Li C., Li Y., Wang S., Zhao X., Zhao Y., Liu Y. (2019). Characteristics of human encephalitis caused by pseudorabies virus: A case series study. Int. J. Infect. Dis..

[104] Wang Y., Nian H., Li Z., Wang W., Wang X., Cui Y. (2019). Human encephalitis complicated with bilateral acute retinal necrosis associated with pseudorabies virus infection: A case report. Int. J. Infect. Dis..

[105] Yang H., Han H., Wang H., Cui Y., Liu H., Ding S. (2019). A Case of Human Viral Encephalitis Caused by Pseudorabies Virus Infection in China. Front. Neurol..

[106] Zheng L., Liu X., Yuan D., Li R., Lu J., Li X., Tian K., Dai E. (2019). Dynamic cerebrospinal fluid analyses of severe pseudorabies encephalitis. Transbound. Emerg. Dis..

[107] Wang D., Tao X., Fei M., Chen J., Guo W., Li P., Wang J. (2020). Human encephalitis caused by pseudorabies virus infection: A case report. J. Neuro Virol..

[108] Fan S., Yuan H., Liu L., Li H., Wang S., Zhao W., Wu Y., Wang P., Hu Y., Han J. (2020). Pseudorabies virus encephalitis in humans: A case series study. J. Neuro Virol..

[109] Hu F., Wang J., Peng X.-Y. (2021). Bilateral Necrotizing Retinitis following Encephalitis Caused by the Pseudorabies Virus Confirmed by Next-Generation Sequencing. Ocul. Immunol. Inflamm..

[110] Ying M., Hu X., Wang M., Cheng X., Zhao B., Tao Y. (2021). Vitritis and retinal vasculitis caused by pseudorabies virus. J. Int. Med. Res..

[111] Yan W., Hu Z., Zhang Y., Wu X., Zhang H. (2021). Case Report: Metagenomic Next-Generation Sequencing for Diagnosis of Human Encephalitis and Endophthalmitis Caused by Pseudorabies Virus. Front. Med..

[112] Zhou Y., Nie C., Wen H., Long Y., Zhou M., Xie Z., Hong D. (2022). Human viral encephalitis associated with suid herpesvirus 1. Neurol. Sci..

[113] Wang J., Cui X., Wang X., Wang W., Gao S., Liu X., Kai Y., Chen C. (2020). Efficacy of the Bartha-K61 vaccine and a gE−/gI−/TK− prototype vaccine against variant porcine pseudorabies virus (vPRV) in piglets with sublethal challenge of vPRV. Res. Vet. Sci..

[114] Ketusing N., Reeves A., Portacci K., Yano T., Olea-Popelka F., Keefe T., Salman M. (2014). Evaluation of Strategies for the Eradication of Pseudorabies Virus (Aujeszky’s Disease) in Commercial Swine Farms in Chiang-Mai and Lampoon Provinces, Thailand, Using a Simulation Disease Spread Model. Transbound. Emerg. Dis..

[115] Ren Q., Ren H., Gu J., Wang J., Jiang L., Gao S. (2022). The Epidemiological Analysis of Pseudorabies Virus and Pathogenicity of the Variant Strain in Shandong Province. Front. Vet. Sci..

[116] Zheng H.-H., Jin Y., Hou C.-Y., Li X.-S., Zhao L., Wang Z.-Y., Chen H.-Y. (2021). Seroprevalence investigation and genetic analysis of pseudorabies virus within pig populations in Henan province of China during 2018–2019. Infect. Genet. Evol..

[117] Lin Y., Tan L., Wang C., He S., Fang L., Wang Z., Zhong Y., Zhang K., Liu D., Yang Q. (2021). Serological Investigation and Genetic Characteristics of Pseudorabies Virus in Hunan Province of China From 2016 to 2020. Front. Vet. Sci..

[118] He W., Zhai X., Su J., Ye R., Zheng Y., Su S. (2019). Antiviral Activity of Germacrone against Pseudorabies Virus in Vitro. Pathogens.

[119] Liu P., Hu D., Yuan L., Lian Z., Yao X., Zhu Z., Nowotny N., Shi Y., Li X. (2021). Meclizine Inhibits Pseudorabies Virus Replication by Interfering with Virus Entry and Release. Front. Microbiol..

[120] Qiu H.-J., Tian Z.-J., Tong G.-Z., Zhou Y.-J., Ni J.-Q., Luo Y.-Z., Cai X.-H. (2005). Protective immunity induced by a recombinant pseudorabies virus expressing the GP5 of porcine reproductive and respiratory syndrome virus in piglets. Vet. Immunol. Immunopathol..

[121] Tian Z.-J., Zhou G.-H., Zheng B.-L., Qiu H.-J., Ni J.-Q., Yang H.-L., Yin X.-N., Hu S.-P., Tong G.-Z. (2006). A recombinant pseudorabies virus encoding the HA gene from H3N2 subtype swine influenza virus protects mice from virulent challenge. Vet. Immunol. Immunopathol..

[122] Zhang C., Guo S., Guo R., Chen S., Zheng Y., Xu M., Wang Z., Liu Y., Wang J. (2021). Identification of four insertion sites for foreign genes in a pseudorabies virus vector. BMC Vet. Res..

