# Supplementary material for: A Review of Pseudorabies Virus Variants: Genomics, Vaccination, Transmission, and Zoonotic Potential
Source: Viruses. 2022 May 9;14(5):1003. doi: 10.3390/v14051003 (PMC9144770; doi:10.3390/v14051003)
Supplement: Supplementary file 1 [file viruses-14-01003-s001.zip › viruses-1716311-supplementary.pdf]

Supplemental Table S1. The genomic information of 39 strains of PRV.

No.	Virus name	Accession No.	Country	Year
1	HNX	KM189912.1	China	2012
2	HNB	KM189914.3	China	2012
3	HN1201	KP722022.1	China	2012
4	JSY7	MT150583.1	China	2018
5	TJ	KJ789182.1	China	2012
6	JSY13	MT157263.1	China	2018
7	HLJ8	KT824771.1	China	2013
8	HeN1	KP098534.1	China	2012
9	JS-2012	KP257591.1	China	2012
10	ZJ01	KM061380.1	China	2012
11	DL14/08	KU360259.1	China	2014
12	LA	KU552118.1	China	1997
13	GD0304	MH582511.1	China	2015
14	Ea	KU315430.1	China	1990
15	Fa	KM189913.1	China	1980s
16	Ea (Hubei)	KX423960.1	China	1993
17	SC	KT809429.1	China	1986
18	HLJ-2013	MK080279.1	China	2013
19	hSD-1/2019	MT468550.1	China	2019
20	GD-YH	MT197597.1	China	2014
21	HuBXY/2018	MT468549.1	China	2018
22	XJ	MW893682.1	China	2015
23	DCD-1	OL639029.1	China	2017
24	JS2019	MW805231.1	China	2019
25	HeNLH/2017	MT775883.1	China	2017
26	SD18	MT949536.1	China	2020
27	Qihe547	KU056477.1	China	2014
28	JX/CH/2016	MK806387.1	China	2016
29	GD1802	MT949535.1	China	2020
30	Becker	JF797219.1	USA	/
31	NIA3	KU900059.1	UK	2008
32	ADV32751/Italy2014	KU198433.1	Italy	2014
33	Bartha	JF797217.1	Hungary	/
34	Kolchis	KT983811.1	Greece	2010
35	Kaplan	JF797218.1	Hungary	2011
36	Kaplan	JQ809328.1	Hungary	2012
37	Kaplan	KJ717942.1	Hungary	2014
38	DUL34Pass	JQ809330.1	Hungary	/
39	DUL34gfp	JQ809329.1	Hungary	/
